# Representational Veracity Domains in Stakeholder Reasoning about iPSC Derivation from Biobanked Samples

**DOI:** 10.21203/rs.3.rs-11324546/v1

**Published:** 2026-10-13

**Authors:** Clement A. Adebamowo, Sally N. Adebamowo, Peter A. Ikhane

## Abstract

Background. We recently derived representational veracity (RV) as an upstream condition for the governance of data science health research: that banked data, the models derived from them and the population descriptors that travel with them continue to represent their sources truthfully at the point of use. The derivation was normative and specified for data; we asked whether the RV domains correspond to anything stakeholders in a real reuse decision recognize, and whether they apply to biological samples. Methods. We conducted a two-stage secondary analysis of the KACIY study, which examined reuse of banked HapMap YRI samples for induced pluripotent stem cell (iPSC) research, using interviews with six community advisory board (CAB) members and five North America-based bioethicists, and two focus groups with twenty community members. We adopted two thematic architectures generated independently before RV existed, then asked how far each corresponds with the four RV domains, returning to the transcripts where needed. An external auditor reviewed the analysis, reading the transcripts before seeing the framework or our conclusions, under responses we specified in advance. Results. Normative authorization and relational community were organizing themes in both architectures and all three groups. Material provenance had a counterpart in the community data only as the legibility of reports returned to the community. Informational descriptors had no counterpart in any community-facing document. No descriptor question had been put to any participant; the fullest descriptor testimony came unprompted from the repository steward. The auditor found descriptor references by five of five bioethicists under a broad definition, three of five under a narrow one, and none by community members. Conclusions. The RV domains are present, unevenly, in stakeholders' reasoning about this reuse decision. The domains concerning persons and a collective were raised by the community that authorized the use; the domains concerning the artifact as it travels largely were not. The audit supported two propositions: that the domains are not equally answerable from every position in the chain of custody and use, and that an RV review should draw on several groups of informants. The reason for the difference was not established, and utility remains to be shown prospectively.

### Background

Research resources in genomics are now banked and used for longer periods, and for more diverse purposes, than those for which they were assembled. A reference panel, a cell line or a genotype file is collected once, under one set of permissions and with one description of the people it represents. It is then distributed, combined with other resources and analyzed with methods that may not have existed when it was created. Each later use relies on information that travels with the resource: where it came from and what has been done to it, how its source population is described, what the donors agreed to, and what relationship the source community continues to have with it. Ethical review of secondary use usually checks whether these records exist and are in order [1–4]. It less often asks whether the records are still true of the resource as it is now being proposed to be used.

This question becomes pressing when a new technology changes what a stored resource can become. Induced pluripotent stem cell (iPSC) methods allow established cell lines to be reprogrammed into stem cells and differentiated into other tissue types [5]. Cell lines that were banked for the study of human genetic variation, such as those of the Yoruba in Ibadan, Nigeria (YRI) panel of the International HapMap Project [6], which have been used widely as a reference resource in genomic research [7], can now be put to uses that neither the donors nor their community could have considered when the samples were collected. The consent, the population label and the community's relationship with the research team all remain attached to these cell lines, but each was established for a different use.

We have recently argued that this question requires a name and an explicit place in ethical review. We call it representational veracity (RV), and we define it as the extent to which banked data, the models derived from them and the population descriptors that travel with them continue to accurately and responsibly represent the biological, social and value-based characteristics of the persons and phenomena they describe, at the point of use and not only at the point of collection [8–10]. RV rests on a persistence-based account of representation, in which a stored datum is a temporal counterpart of its source rather than a fixed object, so that its truthfulness is a relation that can be preserved or lost as the artifact travels. We proposed four domains: material provenance, whether the lineage of the artifact can be reconstructed; informational descriptors, whether labels, categories and population descriptors still track their referents; normative authorization, whether the original permissions still cover the present use; and relational community, whether affected communities retain standing to contest and shape that use [10].

Our derivation of RV was analytical, but it left two questions unanswered. The first is whether the condition that RV names corresponds to anything that people facing a real reuse decision recognize. A framework can be coherent and can be motivated by documented failures elsewhere yet still identify a problem that nobody in the practice it seeks to govern experiences. If that were so here, adding an RV review to the deliberations of institutional review boards, data access committees and biobank oversight bodies would impose a requirement without a corresponding need.

The second question concerns scope, and it is related to how far we can generalize from data to biological samples. We derived the RV framework from considerations of data science use in health research. Its domains were specified for datasets, for the models built from them, and for the population descriptors that accompany them. Its motivating failures are algorithmic, such as a cost variable standing in for health need and a race coefficient that outlived the evidence that once justified it [11, 12]. Extending it to decisions about materials/biological samples, such as whether biobanked samples may be reprogrammed into iPSCs would test this second question. It cannot be assumed that the domains we derived for datasets would necessarily apply to biobanked samples.

Further, we propose that the four RV domains are not equally answerable from every position in a representational chain of data or samples' custody/use, and that a source community, at the chain's origin, is better placed to answer for some of the domains more than others. A community that donated samples stands in two roles at once. Its members gave the original consent/authorization [13], and the community is the collective whose standing in the continuing relationship with data or biological samples is at stake [14, 15]. On these two questions the community is the primary informant, and the documentary record can supplement its account but cannot replace it. The community is also the origin of the material and the referent of the descriptors, but what it can see of the artifact's later travel depends on the engagement maintained with it after collection: the reports that are sent back and the form they take, return visits, and consultation about new uses. We therefore treat the community's view of provenance and descriptors as limited by how the research relationship was designed and maintained, and not as closed in principle.

On this account, we expected community participants to give more weight to the domains concerning persons and the collective, which bear directly on their interests, than to the two domains concerning the artifact as it travels, which lie further from their experience and from what is reported to them. If the proposition holds, then an RV review cannot be conducted with a source community alone, nor with custodians of samples or data alone.

We address these questions using data from the "Knowledge, Attitude, and Recommendations of Community members, Researchers, and Bioethicists on creation of iPSC from YRI HapMap samples" (KACIY) study, in which community members and bioethicists who were involved in the YRI sample collection in 2000 and 2001 were asked, in 2024, whether and how the banked YRI cell lines might ethically be reused for iPSC research. The knowledge, attitudes and recommendations that the study elicited have been published [16]. The study was designed and implemented before we derived the RV framework. Its instruments were built on the benchmarks of ethical research and the principles underpinning the Nigerian National Code for Health Research Ethics, not on any representational construct. We therefore planned our analytic strategy in a way to reduce the risk of finding our own framework in our own data. We began with the thematic architectures that two qualitative data analysts, one of whom was the study's post-doc, generated independently, before RV was formulated and without knowledge of it. Only then did we ask how far each architecture corresponds with the four domains, and where it does not.

## Methods

Design

This is a two-stage secondary analysis of qualitative data from the “Knowledge, Attitude and Recommendations of Community members, Researchers, and Bioethicists on creation of iPSC from YRI HapMap samples” (KACIY) study [16]. In stage one, we adopt two existing framework-blind thematic architectures, and in stage two, we test the correspondence of each architecture with the RV domains. This was supplemented by targeted return to the primary transcripts where the thematic memos could not answer the question. The completed analysis was then reviewed by an independent qualitative analysis auditor.

The prior independent analyses

The parent study generated two independent sets of analytic products before RV was developed, by two qualitative analysts working separately. Neither had access to the RV framework, which did not exist when they worked. Together they constitute two contemporaneous framework-blind readings of this qualitative data.

The first is a set of three thematic memos prepared by the study’s consultant qualitative data analyst (C.D.), who was not involved in the design and implementation of the parent study or in the development of RV. The memos cover the CAB key-informant interviews, the community members’ focus group discussions, and the bioethicists’ key-informant interviews. Each presents verbatim quotations under an inductively generated theme and sub-theme structure [17], with the analyst’s reflections marked as such. They were written in April 2024. The second is a set of two structured analyses prepared by the study’s post-doc (P.A.I.), who conducted the interviews and led the focus groups. The parent study’s analyses were conducted manually and with NVivo. The post-doc’s analysis organized participant responses under a four-theme structure applied identically to the CAB interviews and the focus groups, in paraphrase, and report counts of stated positions. The two architectures are independent of each other, use different theme labels, different sub-theme boundaries and incompatible participant codes, and neither analysis cites the other.

Because the two architectures were generated separately, they permit a stronger test than either alone. Where both coders independently generate a theme corresponding to an RV domain, the correspondence is unlikely to be an artifact of one coder’s interests. Where neither does, the absence is unlikely to be due to coder disinterest.

Reflexive stance

The analytic team includes the investigators who generated these data and the authors of the framework being tested. The lead author was the local investigator for the original YRI sample collection, has maintained the CAB since then, and is the individual in whom participants in the KACIY Study repeatedly expressed trust. The reflexive risk is therefore substantial and runs in a specific direction. Beginning from independently generated architectures is our principal mitigation. We also anchor every claim to verbatim language, report the counts and positions that run against our thesis, and submitted the completed analysis to an external audit, having specified in advance how we would respond to its findings.

Setting, participants and data collection

The source community is in Ibadan, Oyo State, Nigeria. Data collection for the parent KACIY Study ran from November 2023 to May 2024 and covered three groups. Its procedures have been described in full elsewhere [16]. Of the seven surviving members of the CAB that was constituted for the HapMap project, six took part in individual key-informant interviews. Twenty community members took part in two focus group discussions of ten participants each [16]. Random selection from the community was planned, but recruitment was carried out purposively in order to balance each group for sex, age, religion and socioeconomic status.

Five bioethicists based in North America took part in individual key-informant interviews. Four had been involved in the enrolment of participants at the YRI site, and one is the principal investigator of the repository where the samples have been banked since collection, and from where researchers from all over the world request them.

The interview and focus group guides were developed from published work on research ethics and on the ethics of stem cell research and were tested before use with three people who were not members of the study community. They contained no question on population descriptors or on the lineage of the samples. Because CAB and community members might have had limited knowledge of iPSC, an information sheet on iPSC was shared with them before the study began. The post-doc led all interviews and focus groups. A second member of the team was present at each session as note-taker. The lead author attended the CAB interviews virtually without participating and attended the bioethicist interviews as note-taker. Sessions were audio-recorded and transcribed with speech-to-text software (Otter.ai) and manual correction. The study team checked the transcripts for accuracy. Participants were not asked to review them.

Analysis

For stage one we adopted the qualitative data analyst's theme and sub-theme structure as given and consolidated the three memos into a single architecture by grouping sub-themes that appear under different headings in different memos but address the same object. We did not re-theme the corpus.

For stage two, two analysts independently mapped each consolidated theme, from both framework-blind architectures to one of the four RV domains, to more than one domain, or to none, and rated the correspondence as strong, partial or absent. A domain was rated strong only where both framework-blind coders had generated a corresponding theme and its guiding question was substantially the domain’s guiding question. Where the two coders diverged, we report the divergence. Disagreements between the mapping analysts were resolved by returning to the memos and then to the transcripts.

One coding rule was adopted after a review of the first analyst’s coding. Any segment bearing on normative authorization records the referent of the permission under discussion, distinguishing donor consent, institutional ethics committee approval, community permission and repository authorization, so that a general statement about permission elicited by a question about one referent is not reported as a statement about another.

We report counts for the two questions put to every participant in comparable terms. Elsewhere we report the number of groups in which a theme appeared.

External audit

After the analysis was completed, we submitted it to an external qualitative data analyst for audit. Review of a qualitative analysis by a person outside the analytic team is an established means of supporting its confirmability [18]. It is built into consensual qualitative research, in which an auditor checks and challenges the work of the coding team [19], and a formal procedure with defined roles for auditor and auditee has been described and reviewed [20, 21]. We added two features. The first was that the auditor read the primary data before seeing our analysis. The second was that we specified in advance how the manuscript would change in response to each kind of finding she might reach, which applies the reasoning behind preregistration of studies to a qualitative analysis [22].

The auditor (H.O.) is a qualitative analyst with no prior connection to the study, its investigators or the framework. In the first stage she worked from masked transcripts and the interview guides alone. She was asked whether any question about labels or population descriptors had been put to any participant, whether any participant raised such matters unasked, whether the groups received comparable closing invitations, and, where one group raised a topic that another did not, to list and rank the plausible explanations. Her returns were archived before the second stage began. In the second stage she received the manuscript and the framework definitions. She was asked to set out the case against its principal claims, to compare our explanation of the results with her own, to check ten claims against the transcripts, and to record any point on which she disagreed with us. The authors checked the remaining evidentiary claims against the sources. The auditor reviewed the wording in which her views are reported here.

The audit materials are provided in Additional file 1. They comprise our pre-specified responses, the auditor’s assessments, the points on which she continues to disagree with us together with our replies, and the authors’ verification log. The file also describes material that was in the audited draft and that has since been removed for separate publication.

Ethics

The KACIY study was approved by the Centre for Bioethics and Research Ethics Committee, Ibadan, Nigeria (CBR/27/05/2023-26/05/2024) and the Institutional Review Board of the University of Maryland, Baltimore (HP-00107479), and all participants gave written informed consent. This secondary analysis used de-identified material and generated no new participant contact.

### Results

The framework-blind architectures

The qualitative data analyst's three memos consolidated to six themes (Table 1): consent and its adequacy; permission for reuse and who may grant it; uses that must not be permitted; approaching the community, monitoring and building trust; benefits, commercialization and return; and what remained unanswered. All six were present in all three participant groups. The post-doc's two analyses organized the CAB and focus group material under four themes: consent for iPSC research, including a sub-theme on reconsent; rejected uses of samples; benefits from commercializing products from sample use; and engaging communities for future research. The two architectures used different labels and boundaries, and neither cites the other. Two of the analyst's themes, uses that must not be permitted and trust as a foundation of consent, and the post-doc's sub-theme on rejected uses, have no counterpart among the RV domains. They are reported separately.

Table 1
The consolidated framework-blind thematic architecture, generated independently before representational veracity was formulated.

| Theme | Constituent sub-themes across the three memos | Groups in which present |
|---|---|---|
| **A. Consent and its adequacy** | Ideas about giving consent; type of consent used in HapMap; adequacy of the HapMap consent; renewing consent; pros and cons of broad consent; how broad the broad consent should be | CAB, FGD, BE |
| **B. Permission for reuse and who may grant it** | Seeking permission to use leftover samples; what entity may grant it; permission where no advisory board exists; hierarchy of authorities | CAB, FGD, BE |
| **C. Uses that must not be permitted** | Preventing use for unethical goals; iPSC goals supported and not supported; ethical perspective on types of research; remaining concerns | CAB, FGD, BE |
| **D. Approaching the community, monitoring, and building trust** | Ways to approach the community; the advisory board as intermediary; ways to monitor the research; ways to build confidence and trust; the relationship between research team and community; cultural context | CAB, FGD, BE |
| **E. Benefits, commercialization and return** | Companies making profit; sharing benefits and risks; benefits for communities; entities able to distribute products; commercializing outcomes | CAB, FGD, BE |
| **F. What remained unanswered** | Impressions of the questions asked; remaining concerns after interview; questions that remain unanswered; science framework | CAB, FGD, BE |

Correspondence with representational veracity

Table 2 sets out the correspondence between the two architectures and the four domains. Two domains have strong counterparts in both architectures, one has a partial counterpart, and one has no counterpart in either community analysis. We report the domains in order of the strength of their correspondence.

Table 2
Correspondence between two independent framework-blind architectures and the four representational veracity domains.

| RV domain (guiding question) | Proposed informant (a proposition, not a finding) | Counterpart in architecture 1 (qualitative data analyst) | Counterpart in architecture 2 (post-doc) | Correspondence |
|---|---|---|---|---|
| **Normative authorization**: do the original permissions still cover the present use? | The authorizing party, which is the source community | Themes A and B; first or second theme in each memo | Theme 1 entire, including a sub-theme "reconsent to use samples for iPSC research" | **Strong, replicated** |
| **Relational community**: does the community retain standing to contest and shape the use? | The collective whose standing is at issue, which is the source community | Themes D and E; the largest community theme | Themes 3 and 4, "benefits from commercializing products" and "engaging communities for future research" | **Strong, replicated** |
| **Material provenance**: can the lineage of the artifact be reconstructed at the point of use? | Custodians, for chain reconstruction; the source, for legibility at origin | Sub-theme "ways to monitor the complete research"; sample movement filed under consent; bioethicist memo has a monitoring sub-theme drawn from the steward | No monitoring theme; nearest is "making samples available for researchers", which concerns access and not traceability | **Partial**: legibility at source in the community data; reconstruction described by the repository steward |
| **Informational descriptors**: do labels, categories and population descriptors still track their referents? | Those who handle the descriptors downstream of the community | None in any community-facing memo | None; no descriptor codes in either analysis | **None in community data** (no CAB member and no focus group participant). Bioethicists 5 of 5 broad, 3 of 5 narrow (external auditor). Asymmetry unexplained |
| CAB = community advisory board member; FGD = community focus group participant; BE = North America-based genomic research bioethicist. | | | | |

Normative authorization: strong correspondence in both architectures

Theme A and Theme B together constitute the normative authorization domain. The correspondence is exact at the level of the guiding question, which is whether the permission under which the material was entrusted still covers what is now proposed.

Both framework-blind coders generated this theme independently and gave it primacy. Consent is the first or second theme in each of the qualitative data analyst's memos, with the adequacy of the HapMap consent a sub-theme in all three, and it is the post-doc's entire Theme 1 in both of his analyses, where "reconsent to use samples for iPSC research" appears as a sub-theme. It is present in all three participant groups. Without prompting from any framework, it generated the two remedies that RV proposes, time-bounded authorization and re-authorization by a persisting collective.

This is the domain for which the community is the definitive informant, because the community is the authorizing party. The correspondence is correspondingly strong.

Relational community: strong correspondence in both architectures

Theme D and Theme E together constitute the relational community domain. Again, the correspondence is exact at the level of the guiding question, and again both coders generated the theme independently. It is the qualitative data analyst's largest community theme, and it is the post-doc's Themes 3 and 4, "benefits from commercializing products from sample use" and "engaging communities for future research."

This is the second domain for which the community is the definitive informant, because the community is the collective whose standing is at issue. Participants specified the procedures that the domain requires, including a channel for complaint that is independent of the research team.

"as you have come now, if I have to report you, who do I report you to? Mind you, I wouldn't want to report you to Prof. Adebamowo because you all belong to the same research team. I want to confidentially report you to another party … there must be an avenue for that community to feedback … And to be sure that when they feedback, they are heard." (CAB, P5)

Material provenance: a partial counterpart, as legibility at source

Neither framework-blind architecture organizes a theme around reconstructable lineage. The qualitative data analyst's nearest community-facing counterpart is a sub-theme, "ways to monitor the complete research." Her bioethicist memo contains a sub-theme on monitoring the sharing of DNA and cell lines, drawn from the repository steward, and no community-facing document has an equivalent. The post-doc's analyses contain no monitoring theme. His nearest counterpart, "making samples available for researchers and research institutions," concerns access control and not traceability. Material about the movement of the samples abroad appears in the qualitative data analyst's memo under consent and not under custody.

We do not read this as a failure of the domain. What community participants spoke to was what they had seen and what had been sent back to them. They testified to the first transformation, to the loss of visibility when the material left, and to what they could and could not read in the reports sent to them.

“Even when I saw where they packed the samples, the samples are just like this … I saw it; and I wasn’t the only person that saw it … But we saw the samples first before they were taken away.” (CAB, P8)

“At that time, we in the community here did not know that the sample (blood) taken from us will be taken to America; we did not know. We thought they will be kept at UCH … It was later we understood that the blood has been taken to America for other things.” (CAB, P6)

“Is the report just in English or also translated in Yoruba? … The community should be able to make out of that some information other than to see that some DNA sequencing that they may not understand.” (CAB, P5)

The qualitative data analyst’s own comment on the second of these passages concerns communication and confidence and not lineage. She inferred that “some communication problems arose between the community and researchers concerning sample destinations,” and noted that trust in the staff carried the matter.

The community’s interest in provenance is therefore legibility at source: whether the first transformation was witnessed, and whether the report that comes back is in a form that the community can read. We regard this as a distinct component of the domain, and one that a specification written for downstream reviewers does not capture. The auditor did not assess this claim, because she judged that it could not be settled from the material she held. She recorded that her own reading of the transcripts had independently suggested legibility at source as a line of inquiry in its own right. An earlier draft stated that a community cannot speak to lineage because it stands at the first link of the chain and sees none of the rest. The auditor contested that statement, and we withdrew it. What a community can see depends on the engagement maintained with it after collection, through reports, return visits and consultation about new uses. Reconstruction of the chain as a whole was described by the repository steward.

“Any researcher that requests the samples, we review where they are, what institution, what their role is … So that’s the first check we do.” (BE, P5)

Informational descriptors: not raised by community participants

No community-facing document in either framework-blind architecture contains a theme, sub-theme or code concerning labels, categories, ancestry terms or population descriptors. Searches of all four community-facing analytic documents for descriptor vocabulary returned nothing.

Two independent coders working to different designs and generating no descriptor codes converts what might otherwise be an artifact of one coder’s interests into a finding about the data. The auditor’s blind reading of the transcripts agrees. She found no reference to labels, categories, ancestry terms or population descriptors by any CAB member (0 of 6) or by any focus group participant, under either a broad or a narrow definition.

Community participants did speak of the collective that the label names. They offered congratulations to "Yoruba people" and not to individuals, and they assumed that the research team knew what was good for Yoruba land.

"If this thing occurs, I will congratulate them. Congratulations to you people. Congratulation to Yoruba people. We hail them." (CAB, P7)

"To me, you know what is good for Aba-Alamu. You know what is good for Yoruba land. You know what is good for Nigeria." (CAB, P7)

This is the community speaking of itself as the beneficiary and subject of the research. It is not a statement about how the community is labelled in the research record.

Descriptor drift was reported by the repository steward, who handles the descriptors downstream of the community and had acted on them.

"I actually don't like it when there's population genetic projects, and then the genetics get conflated with race and ethnicity … Honestly, when I joined Coriell, the first thing I did was take the superpopulations off the website, because that drove me crazy. And I couldn't find a justification for it anywhere that made sense to me." (BE, P5)

"their entire analysis is structured around these super population groupings, you know, African, European, Asian, and they can't even all agree on who goes where … And every time I say, why did you pick those groupings? How are they helping your analysis? Nobody can ever answer me." (BE, P5)

A second bioethicist described the original project's handling of the population label as a deliberate design decision taken to prevent stigmatizing a named population.

"in order to avoid that kind of labeling, the map said we had contributions from, we oversampled, we anonymized, we sent it to a repository with oversight." (BE, P1)

Among the bioethicists the count depends on the definition, and we report the auditor's counts. Under her broad definition, which admits passing and implicit references, all five bioethicists referred to descriptors. Under her narrow definition, which requires explicit or substantive engagement, three of five did (BE, P2, P3 and P5). Our own reading is narrower. Counting only statements about how the community is labelled or categorized in the research record, we find one report of drift (BE, P5) and one account of the origin of the label (BE, P1). We read the passages she counts for the other two participants, which concern group stigmatization and acknowledgement of the source community in publications, as adjacent to descriptors and not as statements about them. The disagreement concerns where the boundary of the topic lies, and both counts are given so that readers can apply either definition.

We considered whether the community's silence is an artifact of the design. No question about how the community is labelled or categorized in the research record was put to any participant in any of the thirteen interviews, and the guides, which were built on the benchmarks of ethical research, contain no such question. The nearest approach is an interviewer's remark to one bioethicist about the heavy use of the Yoruba samples in ancestry inference, which drew no comment on descriptors. The repository steward introduced the subject herself, in answer to the generic closing invitation to raise anything further, which the post-doc put to her, and the same invitation was extended in both focus groups and in the CAB interviews. The auditor confirmed each of these three points independently.

These facts do not establish why community participants and bioethicists differed. We had attributed the difference to informant position, on the ground that all five bioethicists shared the relevant expertise and the same interviewing, while only the repository steward reported drift. The auditor confirmed the facts but did not accept the inference, because no participant had been asked about descriptors, and because a community that is told how its samples are now labelled could observe a change in the label. Her own explanations, which she listed and ranked before she saw ours, were differential prompting, differences in awareness and expertise, and differences in the information available to participants. As we had specified in advance, we no longer attribute the difference to position alone, and we report it as unexplained.

## Discussion

Do these interviews surface the problems that RV names?

They do, and the framework-blind architectures are the strongest available evidence that they do, because they were generated by two analysts who had never encountered the framework. Two of the four domains are among the themes. Whether a twenty-year-old permission still covers a new use, and whether the community retains standing to be consulted, to monitor, to contest and to benefit, are the organizing questions of four of her six themes across all three memos.

Why the correspondence is uneven: an unexplained asymmetry and four candidate explanations

The asymmetry is not evidence that RV fails in a biospecimen setting but the asymmetry cannot be attributed to any one cause in this study.

Normative authorization and relational community concern persons and a collective. The community is the party that authorized the original use and the collective whose standing is at issue, so it is the definitive informant for both, and both framework-blind coders made both of them organizing themes.

Material provenance and informational descriptors concern the artifact as it travels, and community participants said less about the first and nothing about the second. This observation can be due to several reasons. The first is prompting. No group was asked about descriptors, but interviewers' adjacent questions may have opened the subject more readily for bioethicists than for community participants. The second is expertise. Bioethicists are trained to notice how populations are categorized but populations and their members do not necessarily refer to themselves in the same way. The third is information. Nothing in the transcripts suggests that the community had been told how its samples are labelled downstream, and the quarterly reports that did reach the community did not include population descriptor labels. In any case, such labels were unnecessary as the community knows itself. The fourth is position. The people most likely to use the population labels are those who handle the labels and see them move.

The auditor ranked the first three reasons in that order from her blind reading. We advanced the fourth and continue to regard it as a live hypothesis, because every bioethicist shared the expertise and the interviewing and only the one who curates the labels reported drift. The auditor regards that inference as unverified, and she observes that it answers a question about differences within a group with explanations drawn from differences between groups. We cannot separate the four explanations from this data. The third and the fourth may be one explanation seen from two sides, because a position is in part a matter of what information reaches it. The auditor also added an explanation for the variation among the bioethicists that we had not considered: that the field conceives anonymity at the level of the individual, so that labelling at the level of the population escapes attention. A design that puts descriptor and provenance questions directly to each group, supplies the same information to each, and records what changes would separate these explanations.

Two propositions follow for the framework itself, and the auditor's assessment of them differed.

The first is that material provenance may require a component that a specification written for downstream reviewers omits. At the origin the operative question is whether the source can read the record of the lineage that is returned to it. Whether a reviewer can reconstruct the lineage is a separate question. Community participants asked whether the reports returned to them were in a language they could read and whether they said more than the DNA sequencing they could not interpret. Legibility at source belongs in the domain. The auditor did not assess this claim.

The second is that an RV review should be multi-informant by design. Whatever explains the asymmetry, its practical consequence is the same. Authorization and standing must be assessed with the source community, and cannot be delegated to custodians. Descriptor drift and the reconstructability of the chain were, in this study, described only by those who curate the data. A review that consulted the community alone would have returned two full domains and two empty ones, and might have mistaken the empty domains for absence of concern. If the community's silence reflects what it has been told and not what it could know, the remedy is to route downstream evidence to it and ask again, which strengthens the case for a multi-informant review. The audit supported this proposition, together with the proposition that the domains are not equally answerable from every position.

Does this make RV useful?

Our results establish relevance of the RV framework. The specific work that the framework does in this case can be stated. On this reuse decision the checks that a conventional review applies, to consent, privacy, benefit sharing and community engagement, each returned a clean result, although not every informed reading agrees. A bioethicist read the consent as not allowing iPSC derivation without going back to the community, and the repository has since adopted a policy of declining reprogramming requests for legacy samples. The consent was competently obtained and contains language anticipating unforeseeable risks. Privacy is protected by anonymization and by a repository that releases no identifiers. Benefit sharing was discussed and produced a tangible outcome. Community engagement has been sustained for more than two decades. A conventional review would find nothing to remedy. Participants nevertheless kept returning to something that survives all of these checks: that the material is now doing something the donors did not imagine, under a label that has changed, on the authority of a document written for a different science, with the community's only route of complaint running through the research team. RV gives that concern a name. The auditor did not assess this claim, because she found the checks insufficiently specified in the draft she read, and they are named here in response.

The repository objection must be addressed directly. The steward's account shows a governance regime that is already structured by domain. It tracks provenance, restricts the uses that participants said should be forbidden, reports back to communities, and has adopted a forward policy of declining to approve reprogramming of legacy samples. What that regime lacks is not the checks but their location. It is administered at the point of distribution. Its reports reach the community, but in a form that some members cannot understand, and there is no channel by which the community's reading of them can bear on a decision. RV does not invent the checks. It relocates them so that they are legible and contestable where the representation originates.

Whether an RV review changes decisions is a separate question that these data cannot answer, because no such review was conducted. That requires prospective application in data access committees and oversight bodies, and we regard this as the necessary next step.

### Strengths and limitations

The principal strength of our study is the analytic order combined with independent replication. Two thematic architectures were generated by two coders, working separately and to different designs, before the framework existed, so the correspondence reported here cannot have been produced by our design. Where they converge, on authorization, on community standing and on the absence of descriptors, the result does not depend on either coder's particular interests. The analysis was then audited externally, and we changed our principal interpretation in response. The data also concern a concrete, high-stakes decision and not a hypothetical one, and they include three groups with very different relationships to the material.

The study has several limitations. The analytic team includes the authors of the framework and the investigators of the study, and the lead author, who attended the bioethicist interviews as note-taker, put some of the questions in the interview with the repository steward. Her account of descriptor drift was given in reply to the closing invitation put by the post-doc, and the lead author's comments on the subject followed it. Neither framework-blind analysis was independent of the parent study. The consultant qualitative data analyst was engaged by the study, and the KACIY Study's post-doc conducted the interviews. Their independence is from the framework and not from the investigators. The framework-blind analyses were also not written to answer our question, and three classes of claim could not be made from them: person-level patterns, which theme-organized analysis distributes across sections; prompt sensitivity, since the memos rarely preserve the interviewer's turn; and claims of absence, since a theme missing from a memo may be missing from the data or may simply not have engaged the coder. We returned to the transcripts for each class and have marked those passages. The descriptor result illustrates both the risk and its remedy. The topic was absent from all four community-facing documents in both architectures, which is why we treat the absence as a property of the data and not of one coder, and it was present in the transcripts of the repository steward.

This is a single community with an unusually strong sustained relationship with its investigators, and the authorization finding may present differently where that relationship is weaker. The bioethicist panel is small and deliberately heterogeneous, and we do not claim saturation for it. Participants were never asked whether they recognize the RV description of their concerns, and we make no claim that they would endorse the vocabulary.

Interviewer framing shaped some responses, as reported. The auditor documented leading formulations and interviewer-supplied explanations in several CAB interviews. She also found that in five of the six CAB interviews the interviewers more often characterized iPSC research in general or hypothetical terms than by direct reference to it, whereas the focus groups and the bioethicists were given the explicit term, although CAB and community members had received a written information sheet on iPSC beforehand. Support for continued use among CAB members should be read with that in mind. She observed that focus group respondents answering in turn often repeated one another in closely similar wording, so the focus group tallies may reflect convergence within the group and not independent judgments. The transcripts are edited renderings from which pauses, overlaps and non-verbal cues have been removed.

The audit had limits. The auditor checked ten evidentiary claims against the transcripts, and the authors checked the remainder. She did not assess three claims that she judged to be beyond the material she held. Our pre-specified responses were written before the audit began. They were marked and dated upon receipt.

## Conclusions

We derived representational veracity normatively and for data, and we asked whether the problems it names appear in the reasoning of people facing a real decision about the reuse of biological samples that generate data. Two of its four domains are present in the independent, framework-blind analyses of these interviews as the organizing themes of two architectures generated separately by two coders. The other two are largely absent from the community data. We had explained that by where the community stands in the chain. The external audit did not support that explanation. The reason for the difference remains to be established, and a community's view of how its samples travel and how they are labelled is better described as limited by the engagement maintained with it after collection than as closed in principle.

Two propositions were supported. Material provenance may need a component that a downstream specification omits: whether the record returned to the source is one that the source can understand, in addition to whether a reviewer can reconstruct the lineage. And an RV review should be multi-informant by design, because a review that consulted only the community would, on this evidence, have returned two full domains and two empty ones, and might have mistaken the empty domains for absence of concern.

Participants surfaced these concerns mainly as unresolved tension and not as objection. Most endorsed the reuse, and the same participants who endorsed it said that the meaning of the consent had changed and that they could not follow what became of the material.

Existing review, as it was applied, returned a clean result on this reuse, and the concerns that these participants expressed survive it. Representational veracity gives those concerns a name and indicates where review should look. Whom review should ask, what each informant can see once the evidence is put in front of them, and whether asking improves decisions are the next questions. They require putting the framework, and the questions that this study did not ask, in front of communities, custodians and the bodies that approve reuse.

## Abbreviations

BE
bioethicist
CAB
community advisory board
ELSI
ethical, legal and social implications
FGD
focus group discussion
iPSC
induced pluripotent stem cell
KACIY
Knowledge, Attitude and Recommendations of Community members, Researchers, and Bioethicists on creation of iPSC from YRI HapMap samples
RV
representational veracity
YRI

Yoruba in Ibadan, Nigeria.

### Declarations

**Ethics approval and consent to participate.** The KACIY study was approved by the Centre for Bioethics and Research Ethics Committee, Ibadan, Nigeria (CBR/27/05/2023-26/05/2024) and the Institutional Review Board of the University of Maryland, Baltimore (HP-00107479). All participants signed an informed consent form approved by both committees.

**Consent for publication.** Participants gave written informed consent, which included consent to the publication of de-identified quotations.

**Availability of data and materials.** De-identified excerpts that support the findings appear in the text and tables. The transcripts are not publicly available, in order to protect the confidentiality of participants and of the community but may be requested from the corresponding author under the governance conditions described in this article. Additional file 1 contains our pre-specified responses, her assessments, her remaining disagreements with our replies, and the authors' verification log.

**Clinical trial number.** Not applicable.

**Competing interests.** The authors declare no competing financial interests. Members of the analytic team are investigators on the parent study and authors of the representational veracity framework, and the lead author is the individual in whom community participants expressed trust. These relationships are discussed in the Methods. C.D. prepared the framework-blind thematic memos as the study's qualitative data analyst before the framework was developed, and P.A.I. prepared the second architecture as the study's post-doc. The external audit was conducted by H.O. under a fee that did not depend on her conclusions.

**Funding.** This work was supported by the Knowledge, Attitude and Recommendations of Community members, Researchers, and Bioethicists on creation of iPSC from YRI HapMap samples (ENRICH Project) (Grant number: R25TW011811-S1), the Bridging Gaps in the ELSI of Data Science Health Research in Nigeria (BridgELSI project) (Grant number: U01 MH127693), the Maryland Department of Health's Cigarette Restitution Fund Program (CH-649-CRF), and the University of Maryland Greenebaum Comprehensive Cancer Center Support Grant (P30CA134274).

**Authors' contributions.** The project was conceived by CAA, who was the local researcher for the YRI sample collection and has maintained the CAB to date. PAI, SNA and CAA conducted the study. The manuscript was written by CAA. It was reviewed and approved by all the authors.

**Acknowledgements.** We thank the source community, its community advisory board, the participating bioethicists, and members of the BridgELSI Project which is a part of the DS-I Africa Consortium. We thank Temilola Yusuf, Olusegun Adeyemo and Temidayo O. Ogundiran, who contributed to the conduct of the parent study, C.D., the consultant qualitative analyst, and H.O., who conducted the independent audit.
